# Biofilm-mediated antibiotic tolerance in bacterial pathogens: Integrated molecular networks and novel therapeutic avenues

**DOI:** 10.1080/21505594.2026.2687214

**Published:** 2026-06-11

**Authors:** Wei Zou, Rong Lin, Yanrui Zhao, Peirui Zhan, Xin Zhao, Qian Zhang

**Affiliations:** a Yunnan Provincial Key Laboratory of Public Health and Biosafety & School of Public Health, Kunming Medical University, Kunming, China; b Zhaotong Health Vocational College, Zhaotong, Yunnan, China; c Puai Medical College, Shaoyang University, Shaoyang, China; d Infection Control Office, Xi’an Public Health Center (Xi’an Emergency Medical Center), Xi’an, Shaanxi, China

**Keywords:** Biofilm, antibiotic resistance, antibiotic tolerance, extracellular polymeric substances (EPS), c-di-GMP, quorum sensing

## Abstract

The stable structure of biofilms and the characteristics of the bacteria within them make biofilms an important barrier for bacteria to resist external stress, and a key factor contributing to the difficulty of eradicating clinical infections. This article reviews the multi-stage formation process of biofilms, the various mechanisms of antibiotic tolerance and resistance (such as physical barriers, metabolic adaptations, horizontal gene transfer, etc.), as well as the integrated regulatory roles of molecular networks like quorum sensing (QS) and cyclic diguanosine monophosphate (c-di-GMP). These multiple protective mechanisms in biofilms compose a closed “structure-function” loop system. In the past few years, the emergence of new anti-biofilm intervention approaches (matrix-degrading enzymes, phage therapy, nanomaterials, gene editing, etc.) revealed the possibility to break the limitations of conventional antibiotics by compromising structural integrity or interfering with signaling pathways, providing new ideas for drug-resistance infection control.

## Introduction

Biofilms are composed of microbial communities and their secreted extracellular polymeric substances (EPS), which include polysaccharides, proteins, and extracellular DNA (eDNA) [1–3]. Biofilms are widely distributed and have been found in fresh dairy products [4], around teeth [5], in bones [6], and on medical devices closely associated with hospital-acquired infections [7]. These biofilms derived from distinct anatomical sites and scenarios share common characteristics: microorganisms assemble into stable communities via the extracellular polymeric substance (EPS) matrix and establish local niches conducive to long-term survival and antimicrobial tolerance. Table 1 summarizes common bacteria capable of forming biofilms and the surfaces to which they adhere.

**Table 1.** Common biofilm-forming bacteria and their adhered surfaces.

Gram Stain Characteristic	Bacteria	Surface for Adhesion	Related Diseases or Impacts	Ref.
Gram-positive Bacteria	*Staphylococcus aureus*	Skin, Medical devices	Wound infections, Prosthetic joint infections, Heart valve infections	[8,9]
	*Streptococcus mutans*	Oral cavity	Dental caries, Dental plaque	[10]
	*Enterococcus faecalis*	Heart valves (natural/prosthetic) and medical devices	Endocarditis, Urinary tract infections	[11]
	*Bacillus subtilis*	Plant roots, Bioreactor surfaces	Enhances plant root disease resistance, Improves surfactin production efficiency	[12,13]
Gram-negative Bacteria	*Pseudomonas aeruginosa*	Skin and mucosa, Medical devices	Skin and lung infections, Urinary tract infections, Ventilator-associated pneumonia	[14–16]
	*Escherichia coli*	Food, Dairy processing equipment, Medical devices	Diarrhea, Urinary tract infections, Surgical site infections	[17–19]
	*Vibrio cholerae*	Various surfaces in aquatic environments, Human intestinal epithelium, Immune cell surfaces	Transmission of infection, Kills immune cells	[20,21]
Other Bacteria	*Mycobacterium*	Lungs, Wounds, Medical devices	Lung and skin infections, Iatrogenic infections	[22]

Biofilm-associated microorganisms have been implicated in over 80% of chronic inflammation of soft tissues and infectious diseases, as well as in chronic infections in humans with potential susceptibility [23]. The formation of biofilms on artificial heart valves is a major factor contributing to infective endocarditis, as confirmed by echocardiographic and microbiological diagnostics [9]. In various infectious scenarios, biofilms all exhibit identical core characteristics. Microbes assemble into steady aggregates relying on extracellular polymeric substances, and build localized living microenvironments to sustain prolonged survival and gain resistance against antimicrobial agents.

Unlike free-living planktonic microorganisms, microbial communities within biofilms possess a stable three-dimensional (3D) architecture, which further strengthens specific interspecies crosstalk and cooperative interactions [24]. On this basis, biofilms employ multiple mechanisms including physical barriers, microenvironmental heterogeneity and collective cooperation, rendering the antibiotic resistance of embedded bacteria 10 to 1000-fold higher than that of planktonic counterparts [1]. There are *Klebsiella pneumoniae* strains that are capable of forming biofilm, which are significantly more resistant to antibiotics (*p* < 0.05) than those that are not [25]. *Mycobacterium tuberculosis* markedly increased antibiotic tolerance when forming biofilms. This tolerance arises from the physical barrier function of the biofilm (e.g. the extracellular matrix restricting drug penetration) and the metabolic suppression of bacteria within the biofilm (e.g. dormancy induced by hypoxia or nutrient limitation). Consequently, biofilm-associated infections often become chronic, difficult to eradicate, and prone to relapse [26]. This indicates that the failure of antibiotic treatment associated with biofilms is not attributed to a single factor, but is collectively driven by multiple mechanisms including limited drug penetration, microenvironmental heterogeneity, metabolic inhibition and bacterial collective cooperation. However, the significant evidence may suggest that antibiotic resistance refers to the ability bacteria could acquire through genetic changes – such as mutations or horizontal gene transfer (HGT) – to grow and multiply in the presence of high antibiotic concentrations, with a notable increase in minimum inhibitory concentration (MIC) [27,28]. Moreover, antibiotic tolerance might indicate that bacteria demonstrate the ability to survive temporary exposure to high antibiotic concentrations without changing the MIC, often by slowing or pausing the growth process [27,29]. In addition, antibiotic persistence is characterized by a small subset of phenotypic variants in bacterial populations, termed persister cells. Such cells survive high-dose antibiotic exposure by entering metabolic dormancy or selectively suppressing physiological processes associated with antibiotic targets. This trait occurs without genetic modification and thus does not represent genuine antibiotic resistance [30,31]. Furthermore, the important evidence appears to indicate that HGT mainly helps bacteria acquire and spread resistance, but might not directly cause tolerance or persistence, which could rely on phenotypic regulation and metabolic reprogramming.

In summary, the stable EPS structure and heterogeneous microenvironment within biofilms create a powerful barrier that helps bacteria fight off antibiotics. This not only makes biofilms a critical concern in device-associated infections and chronic infections, but also markedly weakens the efficacy of antibiotics through multiple mechanisms such as antimicrobial resistance and tolerance. It serves as one of the key causes for the persistence, recurrence, and antibiotic treatment failure of chronic infections. That’s why tackling biofilm-related infections remains one of the biggest challenges in clinical infection treatment today.

## Overview of biofilm formation mechanisms

Biofilm creation is a very dynamic, many-stage control procedure containing critical actions such as surface adherence, structural development, and pro-active dispersal. And these process will be precisely regulated by bacterial signaling molecules such as cyclic diguanylate monophosphate (c-di-GMP), and quorum sensing (QS) systems, to form a 3D community with strong drug resistance and environmental adaptability.

### Biofilm life cycle

**Figure 1.**
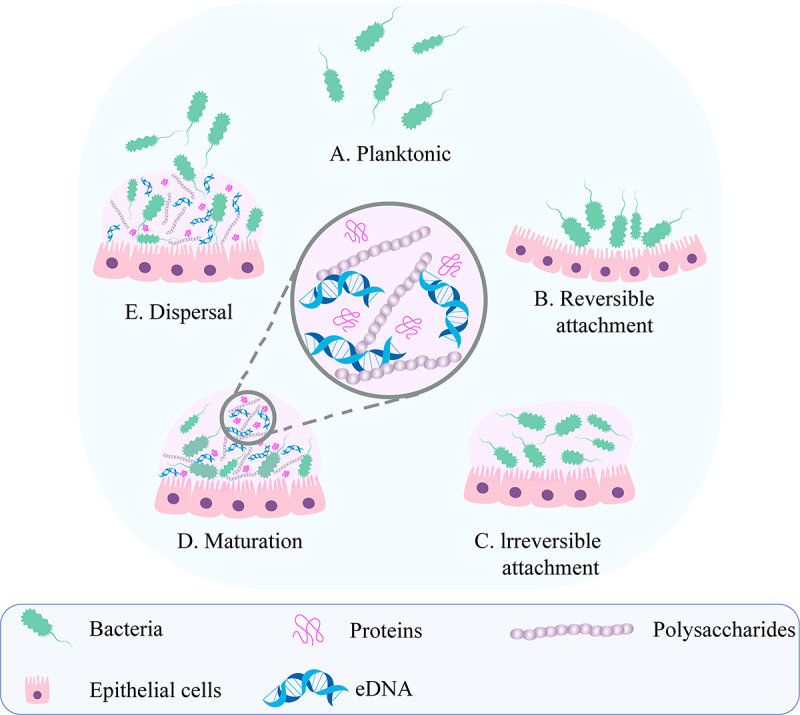
Biofilm life cycle. A biofilm forms in a cycle. First, (A) planktonic bacteria are present. (B) They reversibly attach to a surface through transient contact without stable matrix formation. (C) They irreversibly attach and begin EPS production. Then, the biofilm (D) matures into a 3D structure with channels. Finally, in (E) dispersal, some bacteria are released to start a new cycle of colonization.

Biofilm life cycle starts with planktonic microorganisms (Figure 1(A)), followed by the reversible attachment of these free-floating bacteria to a surface (Figure 1(B)). In the first step, the free-floating bacteria are attached to a surface by physicochemical interactions like van der Waals and electrostatic forces, leading to a first stage of adhesion (Figure 1(B)) [32]. Following reversible attachment, different microorganisms employ specific molecular mechanisms to form stronger adhesion to surfaces or adjacent cells, thereby entering the irreversible attachment phase. In Bartonella henselae, strong adhesion is mainly mediated by the surface-located trimeric autotransporter adhesin Bartonella adhesin A (BadA) [33]. In contrast, several other bacteria like *Staphylococcus aureus* encourage intercellular grouping and microcolonies to form via production of polysaccharide intercellular adhesin (PIA) [34]. These differences indicate that irreversible attachment is not a single unified process, but is collectively driven by specific adhesion strategies adopted by different bacteria according to their own surface structures and matrix components. Microorganisms start to express genes related to biofilm formation during this developmental period. After irreversible binding (Figure 1(C)), the bacterial cells begin to multiply and secrete EPS (e.g. eDNA) to increase surface binding [2,35]. EPS is able to improve the capacity of bacteria to attach onto different surfaces, help bring bacterial cells closer to one another and keep microcolonies staying in a steady condition, and it also functions as the major structural support that contributes to the early-stage development and formation of bacterial biofilms. Within mature biofilms, microcolonies become more structured 3D communities, embedded within an EPS matrix (Figure 1(D)). Within these structures, water channels and pores can be formed, which help nutrients to be exchanged and transported through diffusion and convection [36,37]. This finding indicates that fully developed biofilms are not merely simple gatherings of bacteria. Instead, these collective structures stay in a dynamic state, and they also present uneven spatial distributions as well as the ability to complete internal material transfer and interaction. Some of the bacteria within mature biofilms detach from the biofilm and turn into planktonic states (Figure 1(E)), and this allows them to colonize new surfaces and form a new biofilm. This process is known as biofilm dispersal, which serves as a vital stage in the biofilm life cycle [38]. Figure 1 illustrates the key stages of the biofilm life cycle.

### Key EPS components: polysaccharides, proteins, eDNA, and their functions

Rather than merely being a mixture of polysaccharides, proteins and extracellular DNA, EPS acts as the core component of biofilms and builds up a dynamically integrated structural network. As primary structural elements, polysaccharides mainly consist of glucose, galactose, mannose and amino sugars. These molecules form a three-dimensional framework that grants biofilms mechanical stability and adhesive properties [3,39]. Additionally, as the dominant structural fraction within EPS, polysaccharides are able to form physical and chemical barriers and modulate local microenvironments. This mechanism effectively boosts biofilm tolerance toward antibiotics, host immune attacks and toxic substances [3,40,41]. Changes in polysaccharide content and composition can also affect matrix compactness and molecular diffusion, further influencing overall biofilm functions [42]. In addition, polysaccharides interact extensively with proteins and eDNA within EPS, helping maintain structural stability and functional coordination of the biofilm matrix [43,44]. Mechanistically, polysaccharides act as important structural supporting frameworks. These substances can also adjust the compactness of the extracellular matrix, form barriers that limit substance diffusion and modulate local surrounding microenvironments, and in turn assist biofilms in developing phenotypic traits that enable them to tolerate antimicrobial agents.

Biofilm function may suggest that overall matrix composition and structural organization, rather than individual EPS components, could demonstrate the critical determinants of bacterial community behavior. Notably, studies have shown marked differences in EPS profiles and structural organizations among different bacterial species. For instance, Gram-negative bacteria like *Pseudomonas aeruginosa* use Psl, Pel, and alginate as their key structural polysaccharides, whereas Gram-positive bacteria such as *Staphylococcus aureus* rely on poly-N-acetylglucosamine (PIA) and a variety of proteins for matrix formation [45,46]. Given that the results demonstrate that polysaccharides form the primary structural scaffold in most Gram-negative bacteria, proteins such as lectins and outer membrane vesicle-associated proteins might indicate that cross-linking and structural regulation appear central to matrix integrity, creating a polysaccharide-dominated framework [40,47]. Research shows amyloid proteins dominate matrix organization in some Gram-positive bacteria. Furthermore, the significant evidence may suggest that functional amyloid proteins self-assemble into fibrous structures that could establish the dominant organizational framework in these cases. In light of these findings, polysaccharides might indicate a more supporting role in stabilization and adhesion, resulting in a more protein-centered structural strategy [40,47,48]. This indicates that the extracellular polymeric substance matrix produced by various bacterial strains possesses obvious differences in its main constituent components. Neither its structural features nor functional attributes are decided by a single type of ingredient, and these properties are actually shaped collectively through the mutual interplay among polysaccharides, proteins as well as extracellular DNA.

Evidence shows that these patterns do not hold consistently across different species. However, the relevant findings still indicate that under specific conditions, proteins in some Gram-negative bacteria may also play a dominant structural role [49]; while in certain Gram-positive strains, polysaccharides can similarly form key matrix scaffolds [40]. Although these obvious differences have been observed, these important research results still demonstrate that variations in cell envelope structure, secretion systems, and regulatory pathways can regulate the synthesis of EPS, thereby forming distinct matrix assembly strategies during the evolutionary process. Therefore, this key evidence also lays an important foundation for understanding how different bacterial species support and regulate their biofilm structure.

Proteins present in EPS serve dual roles in biofilm systems, participating in structural formation as well as adaptation to external environments. Previous studies have shown that functional groups on these proteins mediate hydrophobic and electrostatic interactions, which facilitate cross-linking within the extracellular matrix and enhance the adsorption of exogenous compounds [50]. Under environmental stress, microorganisms can modulate protein secretion to improve biofilm stability and tolerance. Examples include elevating surface hydrophobicity or constructing protective layers that alleviate the inhibitory effects of antimicrobial compounds [51,52]. Based on these observations, protein-mediated regulatory mechanisms may also be linked to c-di-GMP signaling cascades, thereby influencing the progression and dispersion of biofilms [53]. This suggests that EPS proteins are not merely simple structural fillers. In fact, these functional substances can simultaneously engage in matrix crosslinking, support environmental adaptation and take part in relevant signal regulation.

As another critical component of EPS, eDNA performs diverse functions in maintaining biofilm structural stability and facilitating developmental progression. It can interact with polysaccharides and proteins to reinforce the structural integrity of the extracellular matrix [3,54,55]. During the early stages of biofilm formation, eDNA also facilitates bacterial adhesion and contributes to microcolony establishment [54,56]. Meanwhile, quorum sensing systems are known to modulate eDNA release and activity as biofilms mature. Programmed cell death and autolysis further mediate eDNA liberation, supporting structural refinement and population homeostasis [57].

Taken together, EPS acts as a dynamic and interconnected network in which polysaccharides, proteins, and eDNA work in synergy to regulate biofilm structure, stress responses, and the penetration of antimicrobial agents. Polysaccharides typically provide the fundamental structural framework of biofilms, whereas proteins and eDNA are mainly responsible for mediating various regulatory and adaptive processes. Interspecies variations in EPS composition also play a key role in determining biofilm stability and the degree of antibiotic tolerance. A deeper understanding of this complex interactive network is therefore crucial for identifying potential targets to combat antibiotic tolerance in biofilm-related infections.

### Regulatory signals: c-di-GMP, QS and related regulatory enzymes

C-di-GMP is a widely conserved second messenger in bacteria, which serves as an important signaling molecule for biofilm formation, stabilization, and dispersal. It helps regulate the transition of bacteria from a free-swimming planktonic life style to a biofilm life style by complicated signaling networks and impacts a variety of biological processes including motility, virulence, and cell cycle progression [58,59]. C-di-GMP effects on biofilm formation by controlling many molecular pathways. It controls adhesins’ localization via Lap system (e.g. LapD and LapG) to decide if bacteria make biofilm [58]. Also, c-di-GMP regulates biofilm-associated pathways through Gac/Rsm, BrlR, and SagS signaling systems [60]. *Pseudomonas aeruginosa* has c-di-GMP levels positively correlated with biofilm formation, particularly in chronic infections, where high c-di-GMP is closely related to biofilm antibiotic resistance and persistence [61]. This finding shows that c-di-GMP works as a central regulatory node to coordinate bacterial adhesion, movement behavior, extracellular matrix synthesis and drug-resistant related phenotypes, instead of only acting as an upstream signal to control a single metabolic pathway alone. C-di-GMP’s key part in biofilm formation makes it a possible goal for treating ongoing illnesses and biofilm-linked sicknesses. For instance, echinacoside reduces c-di-GMP concentrations by targeting c-di-GMP synthases (e.g. SiaD), increasing the effectiveness of antibiotics against biofilm aggregates [62]. These findings reveal that disturbing c-di-GMP metabolism is not just capable of suppressing a single biofilm characteristic. It can reorganize the regulatory balance of bacterial movement, adhesion and matrix synthesis, and consequently increase the sensitivity of biofilms to antimicrobial treatments.

QS is a bacterial cell-to-cell communication process that allows bacteria to coordinate group activities through the secretion and detection of signaling molecules, such as autoinducers [63]. Inside the biofilm, QS is significant for bacterial adhesion, maturation, dispersion, and antibiotic resistance [64,65]. Unlike c-di-GMP that chiefly adjusts the lifestyle shifts of separate bacterial cells, QS places greater emphasis on carrying out coordinated regulation among bacterial populations. In *V.* *cholerae*, small regulatory RNAs (sRNAs) regulate QS signaling pathways that affect the transition from solitary to collective behavior [66]. QS is also capable of modulating the expression of surfactant substances, virulence determinants and genes linked to matrix synthesis, which further exerts influences on the maturation and dispersal progress of biofilms. For *Pseudomonas aeruginosa,* its quorum sensing system facilitates the construction and detachment of biofilm structures by driving the synthesis of rhamnolipid biosurfactants [67]. In methicillin-resistant *Staphylococcus aureus* (MRSA), the upregulation of QS-related genes is closely related to the formation of biofilms and the acquisition of antibiotic resistance [68]. This further proves that the QS system serves as an important regulatory mechanism, which connects the perception of cell density, the coordination of collective bacterial behaviors as well as the formation of drug tolerance traits in biofilms.

Key regulatory enzymes are involved in biofilm formation and regulation by different means like production and degradation of EPS, signal transduction and environmental adaptation. While c-di-GMP and QS are primarily involved in mediating signal regulation, the associated regulatory enzymes are mainly responsible for executive functions, including matrix synthesis, matrix remodeling, and structural maintenance. EPS synthesis is carried out by many enzymes, especially those involved in polysaccharide and protein synthesis. For instance, polysaccharide synthases (such as Wza1 and Wzt, etc.) play essential roles in the synthesis of EPS polysaccharides, indicating that the expression of these enzymes is closely related to biofilm formation [69]. Conversely, destruction of EPS structure or alteration of its physicochemical properties will undermine biofilm stability. Low-concentration ozone is able to target protein chains in EPS and induce their depolymerization, which further impairs the overall biofilm structure [70]. The QS system promotes EPS secretion and biofilm formation by regulating signal molecules such as N-acyl homoserine lactones (AHLs) [71]. C-di-GMP, QS and related regulatory enzymes do not function independently. Instead, they jointly govern the biofilm life cycle from three dimensions: cellular state transition, group behavior coordination and matrix structural execution.

## Mechanisms of biofilm recalcitrance to antibiotics

The remarkable recalcitrance of biofilms to antibiotics is one of the main reasons why clinical infections (such as chronic wound infections and catheter-related infections) and industrial contamination (such as pipeline biofouling, clogging in water treatment systems) are difficult to completely eliminate. Microorganisms within biofilms can withstand antibiotic treatment 10 to 1000 times more effectively than their planktonic counterparts, rendering conventional antibiotic therapies largely ineffective against biofilm-associated infections [1]. Biofilms resist the action of antibiotics through multiple synergistic protective mechanisms, which mainly include the following key processes. From the perspective of action sequence and mechanistic hierarchy, EPS-mediated diffusion limitation and adsorption firstly hinder antibiotic penetration and local distribution within biofilms. Meanwhile, gradients of nutrients, oxygen and pH alter the metabolic state of internal bacteria and reduce the activity of drug targets. Under persistent antimicrobial stress, stress responses and efflux pumps further boost cellular survival and adaptability. Accordingly, biofilm-associated antibiotic resistance should be interpreted as a sequential process consisting of restricted drug penetration, declined target activity and enhanced adaptive defense, rather than the outcome of a single drug-resistant mechanism.

### Physical barriers: the dense structure of EPS hinders antibiotic penetration

**Figure 2.**
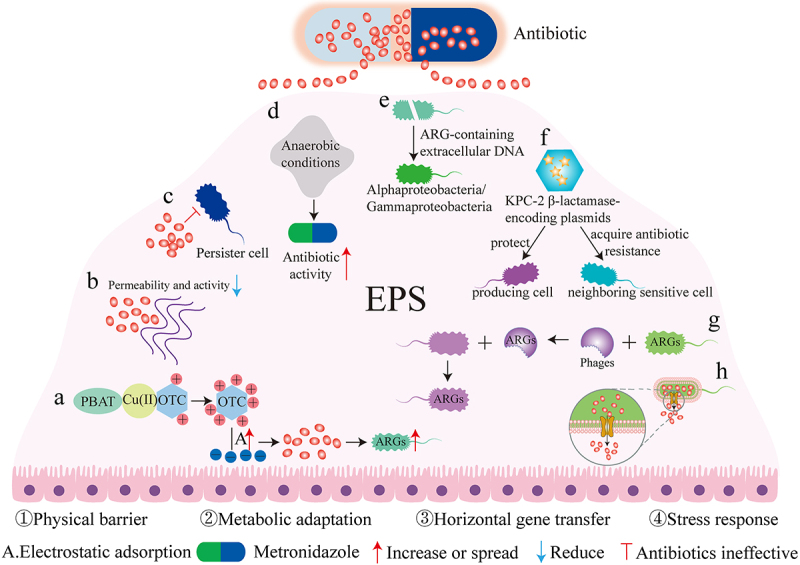
Mechanisms of biofilm recalcitrance to antibiotics. (a) Copper ions (Cu(ii)) form bridging interactions between poly(butylene adipate‑co‑terephthalate) (PBAT) and oxytetracycline (OTC), enhancing electrostatic adsorption of OTC onto microplastics and leading to antibiotic enrichment on the microplastic/biofilm surface, thereby promoting the spread and expression of antibiotic resistance genes(args). (b) The three‑dimensional structure of the extracellular polymeric substances (EPS) matrix creates a physical barrier that reduces antibiotic permeability and activity. (c) Metabolic dormancy in persister cells renders antibiotic targets ineffective. (d) Anaerobic conditions enhance the antibacterial activity of metronidazole. (e) Free DNA released by lysed bacteria is taken up and integrated by neighboring cells, facilitating natural transformation of ARGs. (f) Plasmids encoding the KPC‑2 β‑lactamase carry ARGs, protecting producing cells and conferring resistance on neighboring sensitive cells. (g) Phages transmit ARGs through transduction, enhancing population‑level resistance. (h) Multidrug efflux pumps actively expel antibiotics, reducing intracellular drug concentration and increasing bacterial survival.

The impacts of the EPS barrier on antibiotic efficacy are mainly reflected in two aspects. First, it alters the local distribution of antibiotics on biofilms and carrier surfaces via electrostatic adsorption or molecular binding. Second, its three-dimensional network structure delays the diffusion of drugs into deeper biofilm layers. Among these mechanisms, electrostatic adsorption affects the enrichment and migration of antibiotics at biofilm-related interfaces. Cu (II) forms a link with the surface functional groups of poly (butylene adipate-co-terephthalate) (PBAT) and oxytetracycline (OTC) to enhance the electrostatic attraction between the positively charged OTC and the negatively charged microplastic surface, thus enhancing the antibiotic’s adsorption capacity. This will enhance the adsorption of antibiotics onto microplastics, promoting the transmission and expression of antibiotic resistance genes (ARGs) in the biofilms, thereby exacerbating environmental resistance (Figure 2(a)) [72]. Electrostatic adsorption does not only limit the penetration of antibiotics but also gives the bacteria in the biofilm a “buffer time,” so that they can start to initiate stress response, such as regulating metabolism and activating resistance genes [73]. But it can also enhance the antibacterial action of drugs through electrostatic adsorption. For instance, positively charged rifabutin liposomes adsorb to the surface of negatively charged MRSA biofilms via electrostatic attraction, which can greatly increase the local concentration and antibacterial efficacy of the drug. so it allows for the inhibition of biofilm growth even without penetration [74].

Apart from electrostatic adsorption, the barrier effect of biofilms is also associated with their spatial structure. The 3D network structure of biofilm offers a physical barrier to bacteria, as well as increasing the tolerance of bacteria by decreasing the permeability and activity of antibiotics (Figure 2(b)) [75]. Studies show that the diffusion of antibiotics into biofilms is much slower than in planktonic bacteria, and thus the bacteria in deeper layers have difficulty accessing the antibiotics [76]. EPS polysaccharides and proteins bind antibiotics immobilizing them around the biofilm periphery and thus keeping them out of the biofilm’s interior [77]. The microporous structure of EPS has a significant effect on the metabolic state and antibiotic susceptibility of bacteria in biofilms. This happens because it restricts the penetration of antibiotics and forms concentration gradients of nutrients and oxygen [78]. It is thus evident that the EPS barrier does not merely block antibiotic penetration. Instead, it alters drug distribution via local adsorption and delays drug diffusion into deeper regions through its three-dimensional structure and microenvironmental gradients. Its functional effects may also vary depending on drug properties, carrier charges and surface characteristics of biofilms.

### Metabolic adaptation: hypometabolic state impairs antibiotic-dependent targeting processes

Dormant state of persister cells is believed to be induced by a variety of mechanisms including toxin/antitoxin system, (p)pGpp signal molecules, SOS response, and reduced ATP levels [79,80]. They prevent bacterial metabolism, driving bacteria to a non-growing state that can tolerate the bactericidal effects of antibiotics [81]. Toxins from toxin/antitoxin systems inhibit DNA replication, transcription, or translation, causing dormancy [82]. Hence, the essential role of this low-metabolism state goes beyond simply slowing the proliferation of bacterial cells. It also lowers the activity of multiple physiological processes closely related to antibiotic efficacy, such as cell wall formation, DNA duplication, protein synthesis and energy-driven drug absorption, and ultimately weakens the bactericidal potency of certain antibiotics.

It should be pointed out that persister cells do not form a homogeneous group with complete metabolic dormancy. Though dormancy is their primary feature, a subset of these cells still retain certain metabolic activity when exposed to antibiotic pressure. The transcriptome analysis showed that in *E.* *coli* persister cells treated with high concentration of ampicillin, the expression level of some genes was increased, indicating that persister cells still have metabolism activities [83]. Besides reaching complete dormant status, some persister bacterial strains are also able to enter a slow proliferation state, and they can resume rapid growth soon after antibiotic stress is eliminated [84–86]. This indicates that tolerance induced by low metabolic levels is not restricted to one single dormant form. Instead, it covers a continuous range of states from profound dormancy to relatively weak metabolic activity.

Inside biofilm structures, this kind of metabolic adaptation can be further strengthened by microenvironmental gradient differences. Nutrient shortage, oxygen deficiency and pH variation are commonly observed in the inner region of biofilms. These factors do not act independently, but jointly drive bacteria to shift toward hypometabolic and stress-adaptive states. Under nutrient-deficient conditions, bacteria activate stress response pathways such as RpoS to enhance antibiotic tolerance [87]. Under anaerobic conditions, alterations occur in bacterial metabolic pathways and growth status, which may reduce the activity of certain antibiotics. Accordingly, aminoglycosides such as gentamicin exhibit limited bactericidal effects under anaerobic or low-energy metabolic states. This process is closely associated with electron transport chain activity and proton motive force required for drug uptake [88]. It is clear that the biofilm microenvironment reduces bacterial susceptibility to some antibiotics by remodeling energy metabolism and stress responses.

This mechanism can also explain the distinct bactericidal differences of various antibiotics within biofilms. For instance, ciprofloxacin exhibits significantly higher bactericidal efficiency against planktonic bacteria compared to bacteria within biofilms, which is associated with metabolic arrest in biofilm bacteria preventing antibiotics from functioning effectively (Figure 2(c)) [89]. This phenomenon suggests that biofilm-associated tolerance does not necessarily rely on drug-resistant gene mutations, but can form reversible phenotypic tolerance via metabolic state alterations. Meanwhile, the impact of anaerobic environments on antibiotic activity is drug-specific, rather than simply diminishing the efficacy of all antibiotics. The metronidazole exhibits stronger antibacterial activity in anaerobic environments, as it needs to be reduced to its active form in the absence of oxygen to exert its bactericidal effect (Figure 2(d)) [90,91]. This phenomenon does not negate the mechanism by which hypometabolism promotes tolerance. Instead, it indicates that anaerobic and hypometabolic environments can weaken antibiotics that rely on aerobic respiration, active uptake or vigorous biosynthetic processes, yet may potentiate the efficacy of drugs activated via anaerobic reduction.

Acidic environments can also enhance bacterial tolerance by inducing stress adaptation and altering cellular structures. In acidic environments, bacteria activate specific stress proteins (e.g. universal stress proteins, USPs), which help them adapt to acid stress and enhance tolerance [92]. Low pH also facilitates the utilization of exogenous fatty acids by *Mycobacterium smegmatis*, stabilizing its cell wall and further protecting against antibiotic damage [93]. Therefore, nutrient limitation, anaerobic conditions and acidic pH should not be regarded as separate independent factors, but microenvironmental stresses that jointly act on bacterial metabolic networks. They facilitate the formation of non-heritable and reversible phenotypic tolerance in biofilm bacteria by inhibiting the activity of antibiotic-dependent targeting processes, reshaping energy metabolism and triggering stress adaptation.

### Horizontal gene transfer (HGT): biofilm microenvironments drive ARG dissemination

Horizontal gene transfer (HGT) is one of the primary non‑vertical pathways enabling bacteria to obtain foreign genetic material, and it is crucial for the dissemination of ARGs. HGT mainly occurs through three pathways: transformation, conjugation, and transduction [94,95]. Unlike phenotypic tolerance mediated by hypometabolism or stress adaptation mentioned above, HGT mainly facilitates the acquisition and spread of drug resistance via genetic material transfer. Compared with planktonic cells, the three-dimensional structure and microenvironmental properties of biofilms significantly increase the frequency of HGT. This in turn accelerates the spread of ARGs within microbial communities [94]. This demonstrates that biofilms are capable of enhancing antibiotic tolerance through phenotypic modulation, while also facilitating the spread of heritable resistance via HGT.

Biofilms are composed of densely aggregated bacterial cells embedded within the matrix formed by EPS. Such a structural feature not only shortens the distance between individual bacterial cells, but also provides a stable platform for the exchange of genetic materials. Transformation is significantly enhanced in the biofilm environment. DNA released from lysed bacterial cells can be adsorbed by EPS and stably maintained in the matrix, forming a long-term “DNA reservoir” that increases the likelihood of its uptake by nearby bacterial cells [94]. Members of Alphaproteobacteria and Gammaproteobacteria are capable of taking up this free DNA and integrating it into their own genomes (Figure 2(e)) [96]. In addition, eDNA can form crosslinked structures with polysaccharides, which helps stabilize its presence within the EPS matrix and reduces its degradation by nucleases to a certain extent, thus further improving the efficiency of transformation [97]. This series of processes significantly promotes the continuous spread and accumulation of ARGs inside biofilms.

Conjugation is another major route of HGT. In biofilms, the close physical proximity of bacterial cells significantly enhances the efficiency of plasmid transfer [98,99]. For example, plasmids encoding KPC-2 β-lactamase can spread efficiently within biofilm populations. This not only increases the drug resistance of donor cells, but also confers protection to nearby susceptible bacteria through a “social rescue” effect (Figure 2(f)) [100]. In addition, nutrient limitation and environmental stress in biofilms can upregulate the expression of conjugation-related genes, which further promotes the transfer of drug-resistant plasmids [101]. Some studies have pointed out that the spatial structure of the EPS matrix can restrict cell-to-cell contact in certain contexts, indicating that biofilm structure exerts a dual regulatory effect on conjugation efficiency [102]. Collectively, biofilms exert bidirectional regulatory effects on bacterial conjugation, which are governed by the balance of cellular proximity, plasmid transfer capacity, stress conditions and EPS spatial architecture.

Additionally, transduction, mediated by bacteriophages, could demonstrate that a third pathway for DNA transfer exhibits distinct characteristics in biofilm-associated populations. In light of the evidence, biofilms may suggest that a relatively stable reservoir of host cells supports sustained phage infection and propagation [103,104]. Transduction shows phages package ARGs, enhancing resistance in *Staphylococcus aureus* (Figure 2(g)) [105]. Therefore, the significant spatial heterogeneity of biofilms could indicate that localized infection niches appear to enable repeated phage cycling within confined microenvironments, increasing gene transfer efficiency in specific subpopulations. Notwithstanding these results, the EPS matrix may suggest that phage diffusion appears restricted, leading to spatially heterogeneous transduction dynamics [106,107]. Moreover, such spatial heterogeneity can lead to the localized enrichment of ARGs inside biofilms, further elevating the overall resistance level of the entire bacterial community. The specific contribution of transduction to the spread of ARGs is still not fully understood.

Existing studies have shown that biofilms facilitate HGT through several interrelated pathways, such as increasing cell-to-cell proximity, stabilizing extracellular DNA, and generating stress-adapted microenvironments. However, the significant findings could indicate that these processes collectively appear to increase the efficiency of ARGs spread within microbial communities. These combined mechanisms effectively boost the spread efficiency of ARGs among bacterial, which builds a solid basis for the emergence and spread of heritable drug resistance traits related to biofilms. Targeting HGT processes within biofilms may therefore represent a promising strategy to limit the dissemination of antibiotic resistance.

### Stress responses: adaptive mechanisms strengthen bacterial survival capacity

Bacteria living inside biofilms are constantly subjected to diverse external pressures including antibiotics, oxidative injury, insufficient nutrients and regional oxygen deficiency. To deal with these challenge, bacteria will activate a number of stress response systems such as stringent response, oxidative stress response and SOS response [108,109]. These various response systems work together instead of acting alone. They adjust gene expression levels to improve bacterial adaptability and boost their resistance against antibiotics. This proves that biofilm-related drug tolerance arises from multiple causes, such as limited drug penetration, reduced cell metabolism and active stress adaptation reactions of bacteria.

The stringent response serves as a vital regulatory mechanism, helping bacteria cope with external environmental pressures including nutrient deficiency and antibiotic exposure. Primarily mediated by the signaling molecule (p)ppGpp, it modulates gene expression and redistributes cellular resources, thereby coordinating bacterial growth, metabolism and stress adaptation, facilitating biofilm formation and elevating adaptive antibiotic tolerance [110]. By contrast, the oxidative stress response mainly enables bacteria to resist oxidative damage induced by antibiotics or host immune responses. Mycobacteria can better tolerate oxidants by controlling the production of the KatG enzyme through their oxidative stress response [111]. SOS response is an essential bacterial stress system for DNA damage. Under antibiotic stress, the SOS response induces the expression of DNA repair-related genes to facilitate DNA damage repair and sustain bacterial survival. Meanwhile, its modulation of mutation rates may also improve the capacity of bacteria to adapt to drug pressure. In *Salmonella*, inhibitors targeting the SOS response can markedly reduce bacterial antibiotic tolerance [112]. Mechanistically, the stringent response, oxidative stress response and SOS response jointly strengthen the adaptability of biofilm-embedded bacteria via metabolic regulation, oxidative damage defense and DNA damage repair respectively.

Multidrug efflux pumps act as crucial downstream effector mechanisms by which stress responses boost bacterial drug tolerance and resistance phenotypes. These pumps are protein complexes located on the bacterial cell membrane that actively expel harmful substances such as antibiotics out of the cell, lowering intracellular drug levels and attenuating the bactericidal effects of antibiotics (Figure 2(h)) [113]. In biofilms, stress response systems control efflux pump expression to expel antibiotics from the cell, reducing the killing effect [114]. It is found that oxidative stress markedly enhances *P.* *aeruginosa* efflux pump through induction of the PA5471-MexXY-OprM pathway, thus causing resistance to aminoglycosides [115]. This mechanism shows that efflux pumps are not just basic structures for discharging drugs, but key functional parts involved in bacterial stress adaptation. They also participate in biofilm development and help sustain its structural stability. Studies on *Staphylococcus aureus* have shown that deleting the efflux pump gene norB can change the expression levels of numerous biofilm-related genes and greatly weaken the ability to form biofilms [116]. As a common efflux pump inhibitor, CCCP is able to hinder proton motive force driven drug efflux, and further suppress biofilm development in *Klebsiella pneumoniae*. This finding confirms that efflux pump related pathways take part in maintaining stable biofilm structures [117]. In this whole regulatory pathway, bacterial stress responses carry out overall physiological regulation, while efflux pumps mainly undertake drug removal and biofilm steady maintenance. The cooperation of these two systems greatly improves the survival ability of bacteria under harsh external surroundings.

## Integrative function of molecular regulatory networks

Biofilm development and functional maintenance depend on the dynamic coordination of multi-layered molecular regulatory networks, rather than simple parallel signaling events [118]. From the perspective of systematic regulation, QS, c-di-GMP and stress response systems correspond to three functional levels, namely quorum communication, phenotypic switching and stress adaptation. In the early stage of biofilm formation, QS primarily mediates the coordination of collective bacterial behaviors [119]. As biofilm development proceeds, as a core intracellular signaling molecule, c-di-GMP governs the transition of bacteria from a motile state to an adherent state and promotes matrix synthesis [120]. Notably, QS and c-di-GMP do not act independently; instead, they form an integrated regulatory network through mutual modulation (the relevant mechanisms will be discussed in detail later in this paper). For instance, QS can regulate the expression of enzymes involved in c-di-GMP metabolism, while c-di-GMP can in turn feedback to modulate the activity of QS signaling pathways. Under stress conditions such as antibiotic exposure or oxidative stress, the stress response systems described in ‘Stress Responses: Adaptive mechanisms strengthen bacterial survival capacity’ (e.g. stringent response and oxidative stress response) can remodel QS and c-di-GMP signaling to some extent [118,121]. This regulatory remodeling may further contribute to the reprogramming of cells toward a tolerant and survival-oriented state. This context-dependent hierarchical regulatory mode is a critical basis for biofilm adaptability and the development of antibiotic tolerance [118]. Based on this framework, this section classifies QS, c-di-GMP and stress response systems as the quorum communication layer, phenotypic switching layer and stress adaptation layer respectively, and further discusses the robustness and potential costs of these regulatory networks under perturbations from the perspectives of redundancy and modularity.

### Crosstalk regulation of signaling networks: from biofilm assembly to phenotypic transition

The process of biofilm formation is a dynamic and reversible process that is co-regulated by QS and the secondary messenger c-di-GMP. It allows the whole process, from the initial bacterial adhesion to the final assembly of the biofilm, as well as the transition into a dispersal state. Within this framework, QS and c-di-GMP primarily link quorum communication and phenotypic switching during biofilm development.

**Figure 3.**
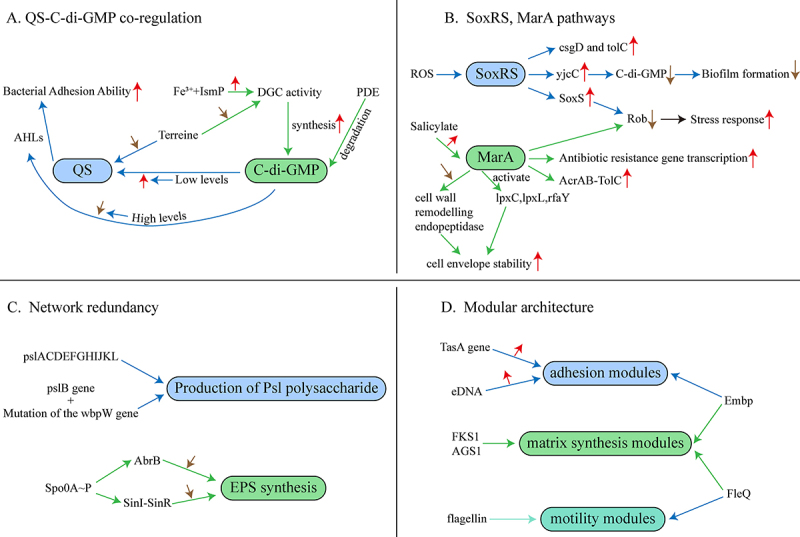
Integrative functions of molecular regulatory networks in biofilms. (A) QS – C-di-GMP co-regulation: QS signals promote surface attachment, while c-di-GMP regulates the transition to biofilm formation; the two systems exhibit bidirectional interactions. (B) Stress response pathways: oxidative stress activates global regulators (e.g. SoxRS, MarA), enhancing antibiotic resistance and efflux activity while modulating biofilm-associated gene expression and c-di-GMP signaling. (C) Network redundancy: parallel regulatory pathways ensure robust EPS production and biofilm stability even when individual components are impaired. (D) Modular architecture: distinct functional modules coordinate adhesion, motility, and extracellular matrix synthesis to support biofilm development.

At the beginning of biofilm formation, bacteria coordinate their adhesion behavior by means of QS signal molecules such as N-acyl homoserine lactones, AHLs. AHLs greatly improve the ability of bacteria to adhere to the surface (such as plastic and pipe material) at first, especially in the range of 10 ng/L-10 μg/L, the promoting effect will increase linearly with the increase of AHL concentration (Figure 3(A)). Moreover, they can induce EPS production, alter EPS hydrophobicity, and activate the transcription of the genes encoding for flagellar assembly, QS and biofilm formation to enhance bacterial adhesion [122]. The functional role of QS signals may shift during the maturation and late stages of biofilm development. In *Desulfovibrio* sp. Huiquan2017, QS signaling molecules such as AI-2 significantly inhibited the late stage of biofilm development, resulting in reduced EPS content and the formation of thinner, looser biofilms with fewer bacterial [123]. QS signal molecules (e.g. AHLs, AI-2, and AIPs) can also promote biofilm fragmentation and bacterial dispersal by activating specific gene expression programs [64]. Accordingly, QS can be regarded as a stage-specific quorum sensing communication system. It not only facilitates bacterial adhesion and biofilm assembly, but also participates in biofilm loosening, fragmentation and dispersal under specific conditions.

The synthesis of c-di-GMP is primarily catalyzed by diguanylate cyclases (DGCs) containing GGDEF domains, which convert two molecules of guanosine triphosphate (GTP) into c-di-GMP [124]. DGC activity is modulated by various environmental stimuli, including light, gases, and metal ions such as iron [125,126]. Iron regulates DGC activity by interacting with the iron-sensing protein IsmP, thereby influencing c-di-GMP synthesis and subsequently affecting *Pseudomonas aeruginosa* biofilm formation and motility (Figure 3(A)) [126]. The degradation of c-di-GMP is mediated by PDEs that contain EAL or HD-GYP domains and convert c-di-GMP into the linear dinucleotide pGpG or GMP (Figure 3(A)) [127]. High c-di-GMP levels generally promote biofilm formation and bacterial sessility. They stimulate the secretion of long-chain, hydrophobic, and neutral extracellular polysaccharides and proteins, while also significantly boosting the bacteria’s capacity for autoaggregation and attachment to negatively charged surfaces [128]. Elevated c-di-GMP levels suppress flagellar motility – for example, in *P.* *aeruginosa*, through MorA-mediated regulation – thereby facilitating bacterial adhesion and biofilm formation [129]. Conversely, low c-di-GMP levels are associated with bacterial motility and a planktonic lifestyle. In *Vibrio cholerae*, low c-di-GMP levels activate the transcription factor TfoY, promoting the transition from an aggregated to a dispersed state and enhancing motility [130]. Within this network, c-di-GMP serves as a core intracellular signal linking environmental inputs to the transition between sessile and motile states, and also participates in matrix production and biofilm dispersal.

Recent evidence indicates that the QS system modulates bacterial physiology by influencing c-di-GMP metabolism. Terreine, for example, simultaneously inhibits QS receptor activity and DGC function in *Pseudomonas aeruginosa*, lowering c-di-GMP levels and consequently suppressing biofilm formation and virulence factor secretion (Figure 3(A)) [131]. On the other hand, c-di-GMP can regulate bacterial community behavior through QS system activity. The high c-di-GMP level in *Pseudomonas fluorescens* inhibits AHLs synthesis, affecting QS and biocontrol (Figure 3(A)) [132]. When the c-di-GMP levels are low, this instead activates pqsR-dependent pathways, which upregulate QS system expression and thus increase the production of virulence factors, which will increase *P.* *aeruginosa*’s overall virulence (Figure 3(A)) [133]. Such bidirectional regulatory relationships do not necessarily jointly promote biofilm formation, and may also modulate virulence, motility and dispersal behaviors.

From a systemic perspective, QS and c-di-GMP together form a signaling regulatory network bridging quorum communication and phenotypic switching. QS mainly converts cell density and population status into collective group behaviors, while c-di-GMP translates environmental and intracellular cues into phenotypic outputs including sessility, motility, matrix synthesis and biofilm dispersal. The crosstalk between the two enables biofilms to shift dynamically among assembly, maturation, dispersal and virulence expression. Such regulatory effects are obviously contingent on bacterial species, developmental stages and external environmental conditions.

### Activation and function of stress-response pathways (SoxRS, MarA) in biofilms

Beyond quorum communication and phenotypic switching mediated by QS and c-di-GMP, the microenvironment inside biofilms frequently presents various stressful conditions. Microorganisms activate specific stress-resistant signaling pathways to sustain their survival. These pathways intertwine closely with biofilm regulatory networks, and modulate microbial adaptability to environmental stress by regulating oxidative stress defense, efflux pump expression, cell envelope homeostasis and biofilm-related gene transcription. Rather than acting as structural components of biofilms, SoxRS and MarA function as key regulatory nodes in stress response. They convert oxidative stress, antimicrobial pressure and environmental chemical signals into altered expression of downstream genes involved in defense response, efflux function, cell envelope integrity and bacterial motility, thereby reshaping biofilm phenotypes.

Among which, ROS is a major physiological response during bacterial stress adaptation, and the SoxRS system plays an important role in this process. Bacteria exposed to ROS (e.g. superoxide or nitric oxide) undergo oxidation of the [2Fe-2S] cluster in SoxR, resulting in conformational changes that activate transcription of SoxS [134]. Inside of biofilms, bacteria are regularly exposed to ROS, and the SoxRS system recognizes these oxidative signals to start defense mechanisms (Figure 3(B)) [135]. It is worth noting that SoxRS exerts no unilateral promotive effect on biofilms, and its influence relies on downstream target genes and local microenvironmental conditions. Study shows that in *Klebsiella pneumoniae*, SoxRS activation lowers c-di-GMP levels by upregulating yjcC to inhibit biofilm formation (Figure 3(B)) [136]. Response regulator FlmD in phosphorylated form interacts with transcriptional activator SoxR to inhibit the expression of acrAB-tolC efflux pump gene thus regulate biofilm formation in Comamonas testosteroni [137]. Mixed species biofilms involving interspecies competition have SoxRS activation that upregulates major genes like csgD which is important for the biofilm matrix production and tolC a mediator of antibiotic efflux/tolerance. These findings suggest that the SoxRS pathway may also play a regulatory role in complex microbial environments (Figure 3(B)) [135]. These findings indicate that SoxRS is better defined as a stress response hub linking oxidative stress, efflux pump regulation and biofilm phenotypic alterations, and its functional orientation is dependent on bacterial species and environmental conditions.

Multiple Antibiotic Resistance Activator (MarA), which belongs to the AraC family and is a transcriptional regulator in *Escherichia coli*, participates in bacterial stress responses and antibiotic resistance regulation [138]. Similar to SoxRS, MarA acts primarily as an upstream transcriptional regulator, indirectly modulating biofilm-associated phenotypes by coordinating multiple downstream functional modules. Structural studies show that immobilizing the N-terminal helix of MarA prevents it from binding to DNA and stops it from activating transcription [139]. The DNA-binding activity of MarA is dependent on 2 HTH motifs and Arg40 in the N- terminal HTH motif plays a role in DNA recognition and tight binding [140]. Environmental chemicals such as salicylate can induce MarA expression, which increases the transcription of antibiotic-resistance-related genes (Figure 3(B)) [141]. Activation of MarA results in the transcription of downstream target genes that are involved in a variety of biological processes, such as the expression of antibiotic efflux pumps, cell envelope processes, and stress responses [138,142]. MarA controls the expression of the AcrAB-TolC efflux pump, the major RND family efflux system in Enterobacteriaceae (Figure 3(B)) [139,143]. It directly activates the LPS biosynthesis genes lpxC, lpxL, rfaY and represses the expression of cell wall remodeling endopeptidases to enhance envelope stability (Figure 3(B)) [138]. In addition, MarA also cooperates with other regulators to repress the expression of genes related to the formation of a biofilm, thereby affecting the bacterial adhesion and environmental endurance [144]. MarA, as an upstream regulator, regulates many different downstream genes, which have different activation and information transmission properties, allowing differential regulation of gene expression under different stress conditions [142,145]. In *Salmonella Typhimurium*, MarA further expands its role in the stress-response network through cross-regulation, where it acts in concert with SoxS to downregulate the expression of Rob (Figure 3(B)) [146]. MarA also regulates motility by inhibiting the expression of flhDC, which results in less flagellum assembly and impaired motility [147]. These results reveal that the effects of MarA on biofilms should be regarded as outcomes of multi-target transcriptional regulation rather than actions through a single pathway.

### Network redundancy and modularity: enhancing system resilience

Biofilm regulatory networks feature obvious redundancy and modularity. Such properties help them retain partial functional stability under external disturbances, and facilitate the development of biofilm persistence and therapeutic resistance. Among these regulatory mechanisms, EPS synthesis and c-di-GMP signaling directly govern biofilm structural formation and state transition. Modules related to adhesion, motility and immune evasion perform stage-specific and context-dependent functions during bacterial attachment, dispersal and host adaptation respectively. Psl polysaccharide synthesis in *Pseudomonas aeruginosa* depends on the psl gene cluster like pslACDEFGHIJKL that plays important roles in Psl biosynthesis and surface adhesion. Mutations in wbpW can be rescued by pslB to restore Psl polysaccharide production, showing functional redundancy between the two genes (Figure 3(C)) [148]. Parallel pathways are designed for a more robust synthesis of EPS; if one fails then there is an alternate pathway for EPS synthesis to continue. In B.subtilis, both the AbrB and SinI-SinR pathways repress EPS production; their respective anti-repressors AbbA and SinI are themselves regulated by Spo0A~P. This parallel design allows Spo0A~P to still activate the SinR pathway through SinI expression when the AbrB pathway fails (Figure 3(C)) [149]. These findings suggest that certain biofilm regulatory networks can enhance systematic robustness via parallel regulation and functional compensation, yet such redundant effects are generally species-specific and condition-dependent.

Signal transduction serves as the core process for biofilms to regulate physiological functions. Its backup mechanisms allow biofilms to retain relevant biological functions even when certain signaling molecules or enzymes are absent. The study shows that the c-di-GMP levels fine-tunes both virulence and biofilm formation of E. amylovora. The PDEs involved in its breakdown are functionally redundant, and the deletion of a single pde gene results in a small increase in c-di-GMP, promoting the development of biofilms; the deletion of multiple pde genes results in excessive accumulation of c-di-GMP, excessively inducing exopolysaccharide synthesis, disrupting the structure of the biofilm, inhibiting motility and type III secretion, and leading to the loss of virulence [150]. This result indicates that redundant signaling pathways do not always strengthen biofilm functions. Imbalanced regulation may also trigger structural abnormalities or bring about virulence-related costs.

Functional modules of biofilms, including adhesion, matrix synthesis and motility modules, maintain relatively independent functions while operating in coordination through limited crosstalk. The adhesion module governs the initial stage of biofilm formation and it is responsible for reversible and irreversible attachment. TasA protein and polysaccharides such as glucan had crucial importance in the primary steps of the attachment in dual species biofilm formation of *Bacillus subtilis* and *Streptococcus mutans*. Mutants devoid of TasA showed less adhesion capacity and thus the polysaccharide-protein adhesion module has a separate function in biofilm formation (Figure 3(D)) [151]. Evidence indicates that eDNA is an independent adhesion module in the biofilm formation of *Burkholderia pseudomallei* biofilms: DNase-induced eDNA removal substantially disrupts initial bacterial attachment and biofilm formation, suggesting that eDNA plays an important role in biofilm formation of this bacterium (Figure 3(D)) [2].

The matrix synthesis module is chiefly responsible for the synthesis and assembly of biofilm extracellular matrix (ECM). ECM embeds microbial cells and sustains the spatial structure of biofilms, which is mainly composed of the aforementioned EPS components. This process is generally accomplished by multiple relatively independent modules, each responsible for the synthesis and assembly of one or several types of matrix components [152]. Researchers have found that *Pseudomonas ogarae* F113 creates its biofilm matrix by creating substances like polysaccharides (Alginate, PNAG), proteins (PsmE, Flp/Tad pili), and this is regulated by certain genes clusters. The deletion of one gene cluster abolishes the production of the corresponding ECM component, but not others [153]. Also in *Candida* biofilms, ECM production and separation are independent and can occur via sonication without damage to the cell wall [154]. ECM synthesis by *Paracoccidioides* is associated with the upregulation of specific genes like FKS1 and AGS1 during the maturation phase of biofilm development (Figure 3(D)) [155]. The motility module is bacteria swimming and spreading, through the flagella and pili. Flagellar protein expression was up-regulated at the early stage of biofilm formation in Desulfovibrio *bizertensis* but down-regulated in the mature biofilm stage, showing that its expression is independently regulated at each distinct stage (Figure 3(D)) [156].

Though these modules function relatively independently, they can coordinate their operation through limited cross-regulation. Besides mediating adhesion of *Staphylococcus epidermidis*, Extracellular matrix binding protein (Embp) also facilitates biofilm formation. Embp contributes to adhesion and matrix-associated biofilm modules (Figure 3(D)), and Embp-driven biofilm formation may impair macrophage phagocytosis and allow immune evasion [157,158]. Furthermore, in *Pseudomonas ogarae* F113, motility is regulated by FleQ, which also controls the synthesis of ECM, suggesting that while the motility module is functionally independent, it still maintains a degree of crosstalk with other modules (Figure 3(D)) [153]. Accordingly, the biofilm regulatory network is composed of multiple relatively independent and interconnected functional modules, rather than being controlled by a single linear pathway. This modular organization improves systematic robustness, while dysregulated regulation may also lead to structural disorders and functional costs.

## Emerging intervention strategies: targeting biofilm structure, regulatory networks and tolerant phenotypes

Traditional antibiotic treatments show poor therapeutic effects on biofilm-related infections. A major reason is that biofilm tolerance typically arises from the combined effects of structural barriers, molecular regulatory networks, and tolerant phenotypes, rather than a single determinant factor. These interactive processes cover physical barriers represented by extracellular polymeric substances, tolerant phenotypes like persister cells, as well as mutual regulatory crosstalk between signaling pathways including quorum sensing and c-di-GMP signaling [159–162]. Notably, such tolerant traits mainly help bacterial communities survive hostile environments, and do not directly strengthen the structural stability of biofilm matrix. Therefore, these interactions should be understood as context-dependent and dynamic, rather than as a universal positive-feedback mechanism. Accordingly, novel anti-biofilm strategies are increasingly designed to target multiple levels of biofilm organization, including structural disruption, signal modulation, delivery optimization, and tolerant-phenotype intervention. These approaches aim to weaken biofilm stability, improve antimicrobial penetration, and enhance the therapeutic outcomes of biofilm-associated infections.

### Targeting EPS and QS systems: disrupting structural integrity and regulatory networks

The recalcitrance of biofilm is not only because of the physical protection of EPS matrix, but also due to the inherent chemical signaling regulation of EPS matrix. From the perspective of mechanistic hierarchy, strategies targeting EPS or extracellular DNA belong mainly to structural-level intervention, while interventions related to c-di-GMP and quorum sensing fall largely into regulatory-level intervention. The two approaches function in distinct ways: one breaks down existing physical barriers, and the other modulates the formation, maturation and dispersal processes of biofilms. Enzymatic degradation method can disrupt the biofilm structure by breaking down EPS and allow the antibiotics and immune cells to penetrate and eliminate the bacteria. For example, MnO2-amylase-PEG-ICG nanosheets(MAPI NSs), which can release α-amylase in the acidicbiofilm microenvironment, greatly degrade MRSA biofilms, and completely clear infections through photothermal therapy [163]. The recombinant SpdAZ (rSpdAZ) isolated from *Streptococcus pyogenes* can degrade the extracellular DNA of biofilm, with strong inhibitory effect (65–93% degradation) on *Pseudomonas aeruginosa* and MRSA in both preventing biofilm formation and disrupting pre-formed mature biofilms [164]. These studies indicate that matrix degradation serves as a direct anti-biofilm strategy, yet its efficacy can still be affected by matrix composition, enzyme delivery efficiency and combined therapeutic regimens.

Interference with the synthesis or breakdown of c-di-GMP can also effectively prevent the formation of biofilms or cause the bacteria to disperse. Unlike direct matrix degradation, c-di-GMP intervention modulates biofilm state transition mainly by regulating motility, adhesion and matrix production. Citrus peel extract from Jeju Island (CPEJ) improves PDE activity, reduces c-di-GMP levels, inhibits biofilm formation and increases bacterial motility [165]. Similarly, Echinacoside reduces the SiaD enzyme activity of *Pseudomonas aeruginosa*, reduces the c-di-GMP level and promotes tobramycin-induced killing of biofilm aggregate [62]. Exogenous c-di-GMP inhibited V. splendidus biofilm formation and disrupted existing biofilms via up-regulation of tricarboxylic acid cycle and nitrogen metabolism, and down-regulation of DNA binding and signal transduction, with biofilm biomass significantly reduced [166]. This also suggests that c-di-GMP-related intervention is not a simple one-way regulation, and its effects can be influenced by bacterial species, biofilm developmental stages and microenvironmental conditions.

Inhibition of the QS system hinders biofilm maturation and improves the efficacy of antibiotics. Compared with EPS or extracellular DNA degradation, quorum sensing inhibition rarely destroys biofilm structure directly. It impairs biofilm stability by weakening bacterial collective behaviors, stress adaptation and tolerance-associated regulation. PH-sensitive curcumin (Cur) loaded clustered nanoparticles effectively penetrate biofilms, suppress QS signal molecule production, and markedly enhance the antibacterial activity of penicillin, ciprofloxacin, and tobramycin [167]. Magnetic nanoparticles(HA@MnFe2O4) down-regulate QS-related genes, such as agrA, agrC, weaken the response to stress, and kill bacteria by hyperthermia [168]. Furthermore, ACR-DMP (a novel photosensitizer) can target bacterial carbohydrates to block QS and two-component signaling systems, and disrupt the function of efflux pumps, which can prevent the recurrence of biofilms [68]. The above strategies are not merely juxtaposed antibacterial approaches, but correspond to distinct functional levels including structural disruption, signal regulation and quorum-sensing mediated collective behavior inhibition.

### Precision antibacterial strategies based on advanced carriers: enhancing drug penetration and targeting persister cells

Conventional antibiotic therapies often fail to effectively penetrate biofilms, resulting in suboptimal treatment outcomes or therapeutic failure [169,170]. Its limited therapeutic efficacy is closely associated with the physical barrier formed by EPS and the presence of internal persister cells within biofilms. All these factors together block drug infiltration, internal diffusion and bactericidal performance [171]. The limited efficacy of traditional treatments is largely attributed to the physical barrier imposed by EPS and the presence of persister cells within biofilms, both of which restrict drug diffusion, distribution, and bactericidal activity [1]. Advanced delivery systems are developed not only to elevate local drug concentrations but also to improve drug penetration into biofilms and help eliminate persister cells, thereby targeting critical nodes in the structure – tolerance loop – including structural barriers and the maintenance of drug tolerance. Carboxylated nanofibrillated cellulose (cNFC) shows considerable potential as a carrier for moxifloxacin, forming a Mox-cNFC delivery system capable of sustained drug release for up to 40 hours. This system can penetrate the biofilm matrix through diffusion, markedly enhancing inhibitory activity against persister cells without changing the MIC [172]. Recent studies have shown that combining aminoglycoside antibiotics with ultrasound-stimulated phase-change contrast agents (US-PCCA) significantly improves bactericidal activity against MRSA biofilms [173]. In addition, host-derived fatty acids such as palmitoleic acid can damage bacterial membrane integrity, thereby promoting gentamicin internalization and helping to overcome biofilm tolerance barriers [174].

However, the significant findings from this study may suggest that different nanocarrier platforms possess important therapeutic characteristics when applied in antibiofilm treatments. In addition to this, the results could also indicate that liposomes, due to their unique amphiphilic structure and excellent biocompatibility, may have the ability to fuse with bacterial membranes and thereby promote the effective delivery of drugs to target sites. Despite these promising results, the available evidence also points to the fact that the relatively poor stability of liposomes, along with their susceptibility to drug leakage, might restrict the overall performance of these delivery systems [175,176]. On the other hand, given that the findings demonstrate superior structural stability, poly(lactic-co-glycolic acid) (PLGA) nanoparticles – which are an FDA-approved material – may exhibit controlled degradation properties as well as sustained drug release capabilities. Specifically, PLGA nanoparticles have been shown to maintain effective drug concentrations at infection sites for an extended period of time [177,178]. Hence, available studies have demonstrated that surface modification of PLGA nanoparticles can facilitate targeted transport, thereby enhancing its application potential in intricate infectious microenvironments. Antibacterial activities of metal-derived nanoparticles like silver and zinc oxide are mainly mediated by multi-target pathways, such as destroying bacterial cell membranes, triggering oxidative stress, and inhibiting the expression of quorum sensing-associated genes [179]. Nevertheless, issues concerning cytotoxicity and biocompatibility remain prominent. In particular, the undefined in vivo metabolic and elimination routes give rise to critical safety risks, which may cause long-term retention in vivo and hinder further clinical translation. Furthermore, related studies have confirmed that silver nanoparticles tend to accumulate in the liver and kidney, and can trigger obvious inflammatory reactions in experimental animals [180]. Based on the above research outcomes, prolonged exposure is likely to result in gut microbiota imbalance and neurotoxicity, which are still major issues requiring attention. At the same time, due to the absence of standardized in vivo evaluation systems, the impacts of particle size and surface modification have not been fully clarified [181]. Furthermore, multi-target intervention can cut down the early occurrence of bacterial drug resistance, yet prolonged treatment exposure still triggers adaptive changes in bacteria. Examples include strengthened antioxidant defense systems and adjusted cell membrane balance, which gradually lower the lasting antibacterial effect. Overall, metallic nanoparticles possess broad application prospects, for they can simultaneously suppress bacterial activity, damage biofilm architecture and interfere with bacterial stress responses. Even so, their biological safety risks and long-term environmental influences still pose key obstacles, greatly limiting their further clinical application and popularization.

Given that evidence demonstrates gene-editing-based precision strategies have introduced new avenues, the results may suggest that targeting biofilm-associated tolerance mechanisms could represent a significant development in the field. The CRISPR-Cas system might indicate that selective disruption of key genes involved in persister formation, such as ptsH in Klebsiella *pneumoniae*, could demonstrate that reducing persister cell populations appears to enhance antibiotic susceptibility [182]. Apart from directly interfering with genes linked to drug tolerance, CRISPR-related techniques can also be used to identify pathogenic bacteria and track the state of persister cells, thus supporting the implementation of precise treatment strategies. Scientists have created a research tool named pSCRATCH. It works by inserting CRISPR sequence labels into bacterial genomes to record the physiological state of persister cells. With this tool, researchers can clearly judge whether clinical treatment failure is caused by the reactivation of dormant persister cells or the emergence of true bacterial drug resistance [183]. Nevertheless, CRISPR strategies face delivery, regulatory, and ethical challenges limiting translation. Therefore, the significant evidence may suggest that off-target effects could indicate that safety concerns appear critical [184], while the results might demonstrate that the lack of efficient in vivo delivery systems remains a key obstacle to the clinical application of these important approaches.

Though the above approaches are effective in optimizing drug delivery and achieving targeted regulation, multiple barriers still restrict their clinical practical use. Major challenges involve the biocompatibility and chronic toxic effects of nanomaterials, unsatisfactory delivery efficiency inside living organisms, plus poor stability within complicated infectious microenvironments. These issues all require more thorough and systematic verification. As for CRISPR-based therapies against biofilms, the unclear regulatory mechanisms of relevant pathways also greatly slow down their progress toward clinical application. At present, there is still no sound and complete regulatory standard system specifically formulated for biofilm-targeted gene editing products. Meanwhile, unified biosafety management norms for the clinical use and environmental release of such gene editing technologies have not yet been established. In addition, delivery carriers including bacteriophages and nanomaterials still need to pass more strict additional safety assessments. All these practical limitations greatly raise the difficulty of putting CRISPR-based anti-biofilm therapies into actual clinical use. Under such circumstances, existing anti-biofilm treatment methods have gradually developed into a multilevel collaborative intervention system, mainly covering biofilm structure destruction, signal pathway regulation and drug delivery system optimization. To systematically clarify the action mechanisms and functional features of these therapies, typical treatment strategies mentioned in ‘Targeting EPS and QS systems: disrupting structural integrity and regulatory networks and Precision antibacterial strategies based on advanced carriers: enhancing drug penetration and targeting persister cells’ and are sorted out in Table 2. These intervention means can regulate biofilm biological characteristics by destroying its complete structure, interfering with intracellular signaling pathways, improving drug delivery efficiency and affecting bacterial drug tolerance related physiological processes.

**Table 2.** Mechanism-based classification of representative antibiofilm intervention and translational strategies.

Intervention Strategy / Mechanistic Layer	Target / Mechanism of Action	Representative Agent / System	Main Antibiofilm Effect	Ref.
EPS Structural Disruption (structural layer)	Degrades extracellular polysaccharides (e.g. starch)	MAPI NSs	Releases amylase in acidic environments, combined with photothermal therapy to eradicate MRSA biofilms	[163]
	Degrades eDNA	rSpdAZ	Degrades eDNA in biofilms of multiple bacteria (*P.* *aeruginosa*, MRSA); inhibits biofilm formation and disrupts mature biofilms (degradation rate 65%-93%)	[164]
c-di-GMP intervention (second messenger level)	Increases PDE activity, reduces c-di-GMP levels	CPEJ	Inhibits biofilm formation and enhances bacterial motility	[165]
	Inhibits SiaD enzyme, reduces c-di-GMP synthesis	Echinacoside	Reduces c-di-GMP levels and enhances the bactericidal effect of tobramycin	[62]
	Metabolic intervention	Exogenous c-di-GMP	Reduces biofilm biomass	[166]
QS intervention (bacterial communication level)	Inhibits QS signal production, enhances antibiotic penetration	Curcumin-loaded pH-sensitive nanoparticles	Enhances the efficacy of multiple antibiotics against biofilms	[167]
	Downregulates QS genes, combined with thermotherapy	HA@MnFe_2_O_4_	Eradicates biofilms and prevents recurrence	[168]
	Inhibits QS and two-component system, disrupts efflux pumps	ACR-DMP	Prevents biofilm recurrence and enhances photodynamic therapy efficacy	[68]
Delivery System Optimization (delivery level)	Controlled drug release, enhanced biofilm penetration	Mox-cNFC	Sustained drug release for 40 hours, effectively inhibits persister cells	[172]
	Ultrasound-enhanced penetration and bactericidal effect	US-PCCA	Significantly enhances bactericidal effect against MRSA biofilms	[173]
	Membrane perturbation promotes antibiotic uptake	Palmitoleic acid and Gentamicin	Perturbs cell membrane, induces antibiotic uptake to overcome resistance	[174]
	High biocompatibility and loading capacity, broad-spectrum antibacterial activity	Liposomal nanocarriers	Effective against Gram-positive and Gram-negative bacterial biofilms	[175,176]
	Controlled release, high loading efficiency, deep penetration	PLGA nanoparticles	Hydrophilicity and negative charge facilitate deep drug penetration into biofilms	[177]
	Interferes with QS, adhesion, and efflux pump genes	Metallic nanoparticles (e.g. Ag, ZnO)	Inhibits biofilm formation and development	[179]
Persister-Targeted Intervention (tolerance phenotype layer)	Knocks out persister-related genes (e.g. ptsH)	CRISPR-Cas system	Significantly reduces persister cell formation in *K.* *pneumoniae*	[182]
Diagnostic and Translational Strategies (translation layer)	Basis for rapid diagnosis and targeted therapy	CRISPR-Cas9 enrichment and Nanopore sequencing	Highly sensitive and specific detection of bacterial infections in blood samples, providing a basis for precise treatment	[185]
	Distinguishes causes of treatment failure	pSCRATCH	Records persister cell state, identifies mechanisms of treatment failure	[183]

### AI-driven high-throughput drug screening: developing biofilm-specific inhibitors

Artificial intelligence (AI) – especially machine learning (ML) and deep learning methods – is bringing about a clear shift away from traditional biofilm detection tools, moving toward platforms that aid in the discovery and optimization of antibiofilm agents. Unlike the conventional empirical approach to drug discovery, AI makes it possible to efficiently prioritize candidate molecules by learning the relationships between structure and function from existing datasets. This capability helps researchers identify compounds that specifically target biofilms. Some commonly used models – such as random forest, support vector machines (SVM), and deep neural networks – all perform well in predicting outcomes during antimicrobial and antibiofilm screening. Together, these AI approaches lay a solid foundation for speeding up the overall discovery process. What’s more, both machine learning and deep learning models have proven to be practically useful in natural product research too. From the perspective of action principle, artificial intelligence cannot directly act on biofilms. Instead, it offers reliable technical methods to carry out accurate anti-biofilm intervention, speeding up candidate drug screening, biological activity prediction as well as the optimization of drug delivery systems.

In actual research practice, random forest and deep learning models are widely applied to screen natural active substances. By learning and analyzing molecular structure features to complete predictive analysis, these models can quickly filter out potential anti-biofilm active molecules from massive compound databases. To give an example, a machine learning model that analyzed 61 essential oils was able to predict antibiofilm activity against *Pseudomonas aeruginosa* strain 37P with an accuracy of 88% and an MCC value of 0.71. Through this analysis, the model successfully identified key active compounds like linalool and eucalyptol [186]. When it comes to antimicrobial peptide design, combining deep learning with sequence optimization algorithms allows researchers to build models that predict peptide activity – this in turn makes it easier to screen and refine potential peptides quickly. One notable example is CIT-8, a peptide optimized using machine learning. This peptide reduced the number of viable cells in mature MRSA and VRSA biofilms by 3 log_1__0_ and 4 log_1__0_, respectively, which translates to 99.9% and 99.99% clearance rates. What’s more, it completely eradicated persister cells (around 1 × 10^8^ CFU) within just 30 minutes [187]. Related research has confirmed that artificial intelligence can markedly boost the screening speed of potential anti-biofilm molecules, and also provide reasonable guidance for molecular modification aimed at persister cells and fully formed biofilms.

AI’s applications extend far beyond molecular discovery; it is also widely used to optimize drug delivery systems and formulation performance. For example, using an artificial neural network (ANN) model to optimize chitosan nanoparticle synthesis, the actual yield under optimal conditions was 21.15 mg/mL, which is highly consistent with the ANN-predicted 20.21 mg/mL. At 1500 μg/mL, these nanoparticles inhibited the biofilms of *Pseudomonas aeruginosa*, *Staphylococcus aureus*, and *Candida albicans* by 75.96%, 35.81%, and 67.86%, respectively [188]. Similarly, AI-driven models based on random forest algorithms achieved over 80% accuracy in identifying unknown clinical isolates of biofilm-forming pathogens. Guided by these models, antibiotic-loaded cocktail nanoprobes can penetrate biofilms for efficient eradication [189]. These studies show AI applies not only to candidate molecule discovery but also to drug delivery system optimization.

Notwithstanding these advances, AI-based approaches still have several important limitations. AI models’ performance relies heavily on large, high-quality datasets – and such datasets are still relatively scarce in biofilm research [190]. AI-driven nanomaterial screening does offer clear benefits in early-stage development, but its clinical translation is held back by high manufacturing costs, strict regulatory demands, and complex standardized validation processes [191]. AI predictions still need to be validated through in vitro and in vivo experiments, and we haven’t fully established their cost-effectiveness or scalability in routine clinical settings. What’s more, candidate molecules found via AI might still pose risks of triggering resistance or adaptive evolution when used clinically. That’s why their long-term efficacy and ecological impact on microbial communities call for further careful evaluation.

In general, nano drug delivery systems, CRISPR intervention strategies and AI-assisted molecule screening fully reveal the overall development trend of anti-biofilm therapy. This field is gradually shifting from traditional empirical medication mode to accurate and systematic intervention treatment. Nevertheless, the clinical translation of these emerging strategies is restricted by multiple factors. The complex biofilm structure impedes the effective penetration and distribution of drugs and delivery systems. There is a lack of standardized evaluation criteria for the in vivo toxicity of metallic nanoparticles. CRISPR-based therapies are hindered by incomplete regulatory frameworks and immature biological containment strategies, while the cost-effectiveness and practical accessibility of AI-driven screening have not been verified in routine clinical practice. In addition, most strategies remain at the stage of conceptual verification and animal experiments, and their long-term safety, drug resistance evolution risks and clinical applicability in complex chronic infection models require further systematic research. Persistent drug selective pressure can induce bacteria to develop novel tolerant phenotypes, promote drug resistance evolution and bacterial adaptive reprogramming, and further weaken the therapeutic effect of long-term intervention.

## Summary and outlook

Biofilms are a key survival technique used by bacteria in unfavorable surroundings. And its very complicated structuring as well as functioning combination is helping a lot the bacteria against the antibacterial products that are being given out in hospitals to get rid of it for patients. High antibiotic tolerance of biofilms is not caused by one factor, it is the result of a variety of coupling among structure and function. On one hand, the EPS matrix forms a physical barrier to restrict antibiotic penetration. Instead, differences in the environment inside of a biofilm lead to different metabolic states that bacteria must adopt, leading to persister cells. And then signaling molecules like QS factors and c-di-GMP that control gene expression and phenotypic plasticity can also stabilize the biofilm structure and tolerance. All these mechanisms are present as synergistically acting and highly redundant, but modular regulatory networks, which provide a robust “structure-function” feedback system, bestowing survival advantages to the bacteria under antibiotic stress.

Though we have made considerable headway in understanding the underlying mechanisms of tolerance at the molecular level, there are still three major bottlenecks for clinical translation: (1) the current interventions, such as matrix-degrading enzymes, often target only one pathway, whereas the modular biofilm network compensates through alternative pathways; (2) the clinical safety and delivery efficiency of nanoparticle-based and CRISPR-Cas antibacterial therapies need to be optimized; (3) the accuracy of AI-driven personalized biofilm infection treatments needs to be improved. In the future, researchers should incorporate multi-omics technologies like transcriptomics, proteomics, metabolomics, and spatial omics to understand the dynamic regulatory networks that govern biofilm formation and tolerance at the systems level. Developing in vivo biofilm models that more closely mimic the human infection environment, such as organoid co-culture and humanized animal models, will increase the translational feasibility. Also, using AI to predict the susceptibility to biofilms, to design intelligent nanoparticle-based drug delivery systems, to disrupt biofilm structure and reset bacterial metabolism, will lead to break the feedback loop of tolerance of biofilms.

## Data Availability

Data sharing is not applicable to this article as no new data were created or analyzed in this study.
